# Effects of *Ginkgo biloba* on Endurance and Cognitive Performance in Male Mountaineers Under Normobaric Hypoxia After Acetazolamide Treatment

**DOI:** 10.1002/fsn3.72290

**Published:** 2026-08-23

**Authors:** Sara Mansouri, Vahid Tadibi

**Affiliations:** ^1^ Exercise Metabolism and Performance Lab (EMPL), Department of Exercise Physiology, Faculty of Sport Sciences Razi University Kermanshah Iran

**Keywords:** acute mountain sickness, altitude, cognitive function, mountaineering, reaction time, time to exhaustion

## Abstract

This study examined whether *Ginkgo biloba* (GB), used as an adjunct to acetazolamide (Az) for acute mountain sickness (AMS) prevention, affected endurance and cognitive performance under normobaric hypoxia in male mountaineers. This randomized, double‐blind, placebo‐controlled crossover trial included four sessions and two supplementation phases (GB and placebo) separated by a 1‐week washout period. Testing was conducted under normobaric hypoxic conditions (13% O_2_). Twelve healthy male mountaineers (aged 18–44) with prior high‐altitude experience (above 4000 m), a history of AMS, and regular aerobic training were included. Participants received GB (200 mg/day) or a placebo for 5 days, with Az (125 mg, three doses) administered during the final 2 days of each phase. GB supplementation was associated with a significantly longer time to exhaustion (*p* = 0.019) and lower ratings of perceived exertion (*p* = 0.020) during treadmill exercise to exhaustion under hypoxia. However, no significant between‐condition differences were observed for the Feeling Scale, Felt Arousal Scale, heart rate, or peripheral oxygen saturation during the endurance test. No significant differences were found in choice reaction time or Stroop Color‐Word Test outcomes. In conclusion, GB supplementation for 5 days was associated with a longer time to exhaustion under simulated altitude in male mountaineers using Az for AMS prevention, while no significant between‐condition differences were observed in cognitive outcomes. The endurance finding is preliminary and should be regarded as exploratory pending confirmation in larger studies. Because Az was administered in both conditions, the effect of GB without Az cannot be determined from this design.

**Trial Registration:** The trial was registered in the Iranian Clinical Trial Registry (www.irct.behdasht.gov.ir, IRCT ID: IRCT20230711058744N1)

## Graphical abstract

Among male mountaineers receiving acetazolamide prophylaxis for acute mountain sickness, 5 days of *Ginkgo biloba* supplementation was associated with longer time to exhaustion and lower perceived exertion during treadmill exercise under normobaric hypoxia. No significant differences were observed in heart rate, peripheral oxygen saturation, affective responses, or cognitive performance.

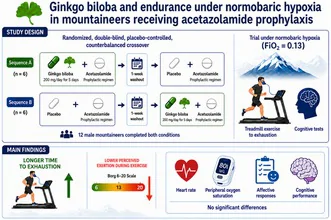

## 1 Introduction

At high altitudes, the partial pressure of oxygen decreases, leading to reduced oxygen availability and hypoxia (Berger and Luks 2023; Li et al. 2024). Acute mountain sickness (AMS), which includes symptoms like headache, dizziness, fatigue, and nausea, can occur when ascending above 2500 m without proper acclimatization (Luks et al. 2024). Acetazolamide (Az), a carbonic anhydrase inhibitor, is recommended for the prevention of AMS and is most effective when initiated before ascent (Luks et al. 2024). A systematic review and meta‐analysis have confirmed its significant efficacy in preventing AMS (Gao et al. 2021). The recommended adult prophylactic regimen is 125 mg every 12 h, beginning the day before ascent (Luks et al. 2024). However, Az may cause metabolic acidosis by increasing renal bicarbonate loss, which in turn stimulates ventilation (Luks et al. 2024). A recent study suggests that Az can impair exercise performance during short‐term hypoxia exposure (Chang et al. 2024). High‐altitude hypoxia can also adversely affect cognitive performance; for example, recent evidence has shown impairments in concentration and memory at high altitude (Chen et al. 2023), and diminished cognitive function under hypoxic conditions (Ramírez‐delaCruz et al. 2024). Furthermore, Az has been linked to impairments in cognitive performance shortly after ascent, although evidence regarding its cognitive effects at high altitude remains mixed (Phillips et al. 2017; Reiser et al. 2023; Wang et al. 2013).

GB is an herbal extract derived from the leaves of the maidenhair tree, a species native to China (Zhang et al. 2023). This unique tree species has been used in traditional medicine for centuries and is rich in biologically active compounds, particularly flavonoids and terpenoids, which contribute to its therapeutic effects (Akaberi et al. 2023; Zonouz et al. 2024). Research suggests that GB can enhance cognitive function by regulating cerebral blood flow and antagonizing platelet‐activating factors, thereby helping protect the brain from ischemic injury (Cui et al. 2023; Zonouz et al. 2024). Recent evidence also suggests that GB may improve endurance‐related outcomes and may alleviate angina symptoms and myocardial ischemia in patients with unstable angina (Liu et al. 2024; Shahrajabian and Sun 2025). Additionally, GB has been considered a potential agent in the context of AMS because of proposed mechanisms such as reducing tissue hypoxia, promoting vasodilation, and decreasing oxidative stress (Akaberi et al. 2023; Silva and Martins 2022). Thus, GB may be a potential option for influencing cognitive and endurance‐related responses under hypoxic conditions. In the present study, Az was included to reflect its prophylactic use during a simulated acute ascent in mountaineers with a history of AMS and without recent altitude acclimatization. Given the potential adverse effects of Az and the possible benefits of GB on endurance and cognition, this study examined whether GB, used as an adjunct to Az for AMS prevention, affected endurance and cognitive performance under normobaric hypoxia in male mountaineers. Accordingly, we hypothesized that, compared with placebo under the same Az regimen, GB would prolong time to exhaustion under acute hypoxia and better preserve cognitive performance during hypoxia and after exhaustive exercise. HR, SpO_2_, and perceptual responses were included as accompanying measures during the endurance test rather than as tests of a specific mechanism. This study is especially pertinent due to the rising number of people participating in high‐altitude activities and the need for effective strategies to address altitude‐related health issues.

## 2 Materials and Methods

### 2.1 Study Design

This study employed a randomized, double‐blind, placebo‐controlled crossover design with four laboratory sessions. Each participant completed a GB phase and a placebo phase, with Az administered according to the same schedule in both phases.

### 2.2 Participants

Twelve healthy male mountaineers participated in the study. Inclusion criteria required participants to be aged 18–44, experienced climbers who had ascended above 4000 m, and had engaged in regular mountaineering in the past year. They also needed a history of AMS, a BMI between 18.5 and 24.9, and general health for physical activity. Additionally, participants had to engage in regular aerobic exercise for at least 6 months, with a minimum of three sessions per week. This criterion was intended to recruit physically active mountaineers accustomed to sustained aerobic exercise and to reduce the likelihood that low general exercise tolerance would determine termination of the endurance test. Exclusion criteria for study entry included chronic disease, histories of cognitive or neurological disorders, allergies, the use of sports supplements within 2 months before the study, smoking or alcohol consumption, blood donation within 2 months before the study, overnight stays above 2700 m or taking Az in the 2 weeks leading up to the study, allergies to Az or sulfonamides, and color blindness. Withdrawal criteria included inability to complete the study for any reason, illness or injury during the study, discomfort with the hypoxic intervention (mask removal), oxygen saturation dropping below 65%, or reporting dizziness or chest pain during tests.

**FIGURE 1:**
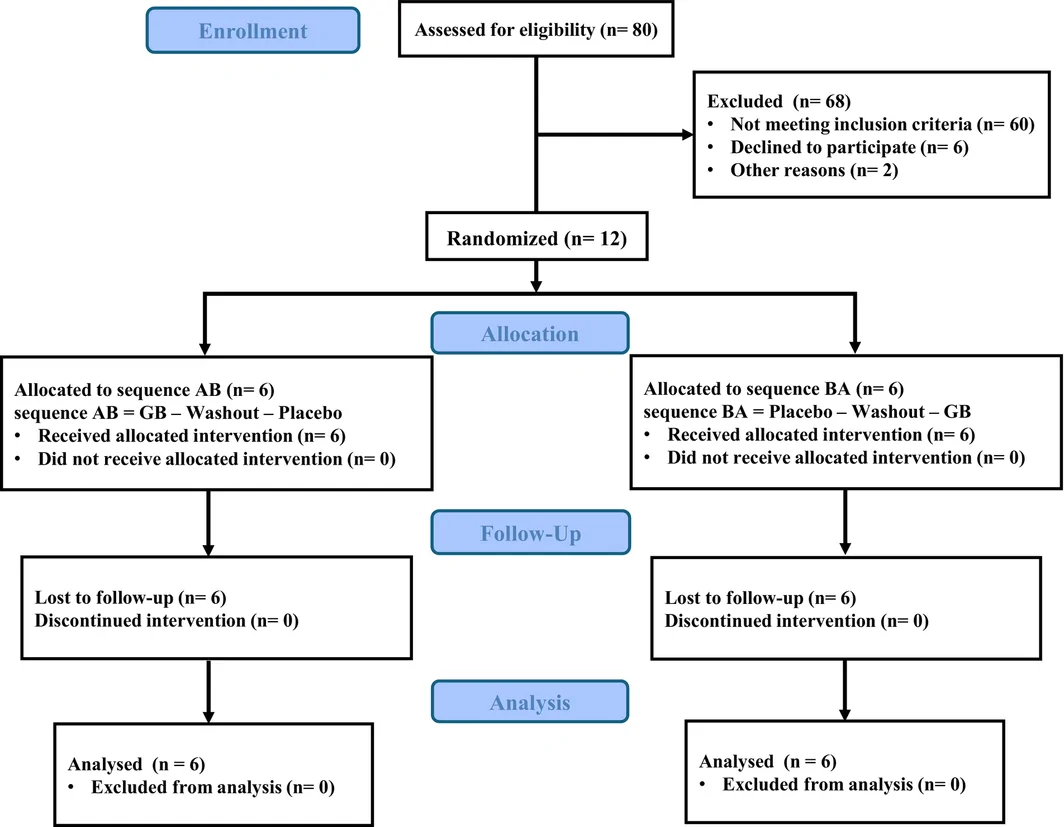
Participants flow.

The flow of participants through the study is illustrated in Figure 1.

### 2.3 Testing Procedure

During the initial laboratory visit, participants were informed about the research methodology and tests, provided written consent, and received essential information and recommendations. A 125 mg dose of Az was administered to assess potential adverse reactions and a laboratory attendance schedule was established.

In the second visit, participants underwent anthropometric measurements, familiarization with the hypoxia device, and cognitive and perceptual function tests. They then performed an incremental treadmill test to determine their maximum aerobic speed (MAS). A 24‐h dietary recall was conducted through interviews, and participants were instructed to maintain the same diet leading up to the following sessions. An independent researcher used Random Allocation Software to assign participants by block randomization to one of two counterbalanced supplementation sequences: GB followed by placebo or placebo followed by GB, with a 1‐week washout period between conditions.

During the third and fourth visits, after 5 days' administration of GB or placebo, participants completed cognitive tests, including the Stroop Color Word Test (SCWT) and choice reaction time (CRT) tasks, in the laboratory. They then underwent 60 min of normobaric hypoxia (O_2_ = 13%, simulating ~3800 m altitude) while resting, with heart rate (HR) and blood oxygen saturation (SpO_2_) monitored and recorded every 10 min. After normobaric hypoxia exposure, the cognitive tests were repeated, followed by a submaximal running test to exhaustion at 70% of the previously measured MAS. During the time to exhaustion, HR, SpO_2_, and perceptual responses, including the rate of perceived exertion (RPE), affective valence, and perceived activation were recorded every 3 min. The cognitive tests were repeated after exhaustion. To minimize inter‐individual variability, participants were tested at the same time of day under standardized conditions and were instructed to maintain the same diet across sessions, avoid strenuous physical activity before testing, and to refrain from caffeine, alcohol, and energy drinks on testing days. In addition, the randomized, double‐blind, placebo‐controlled crossover design with a 1‐week washout period was used to reduce variability across conditions.

### 2.4 Az Administration

The Az used in the study was obtained from the Iran Daru Company (product code: 06260155400092, Iran), with its authenticity confirmed via an online source (ICR.fda.gov.ir). Participants were instructed to take a total of three doses of 125 mg each during the two GB and placebo administrations. This regimen included taking two pills on the day prior to the testing, which were to be consumed after breakfast and then again after dinner. Additionally, participants were required to take one final pill on the actual test day, again after having breakfast.

### 2.5 GB and Placebo Administration

GB capsules were sourced from Jam Tadbir Kala Pharmaceutical Company (product code: 06260589800895, Iran), under license from Laboratories Vitarmonyl, France, with authenticity confirmed by the Food and Drug Organization through the TTAC website. Manufacturer‐reported product information indicated that each hard capsule contained 200 mg of a concentrated, titrated *Ginkgo biloba* extract, including 2 mg terpene lactones and 8 mg flavonol glycosides. Participants were instructed to take one capsule each morning with water for five consecutive days. The selected dose of 200 mg/day was considered appropriate because it falls within the adult oral dosing range described for standardized GB extracts in regulatory monographs (European Medicines Agency‐Committee on Herbal Medicinal Products 2015). Placebo capsules contained maltodextrin and were prepared to match the GB capsules in appearance. Participants and investigators involved in testing remained unaware of the condition assignments. At the end of each supplementation phase, before any information regarding allocation was disclosed, participants were asked whether they believed they had received GB, placebo, or were uncertain.

### 2.6 Incremental Running Test to Determine Maximal Aerobic Speed

An incremental running test was conducted on a treadmill (h/p/cosmos, Germany) to determine MAS. Participants began with a 3‐min warm‐up at 6 km/h and a 1% incline. The test then commenced at 8 km/h with a 1% incline, increasing by 1 km/h every 3 min. Verbal encouragement was provided to maximize effort. Ultimately, the calculation for the MAS was derived from the final speed that the participant completed successfully alongside the duration of the final stage that was not fully completed (Machado et al. 2013).

### 2.7 Submaximal Running Test to Exhaustion

Participants performed a submaximal running test by warming up on a treadmill (h/p/cosmos, Germany) and then running at 70% of their previously determined MAS until exhaustion under normobaric hypoxic condition. Exhaustion was indicated by an inability to maintain the prescribed speed, a heart rate exceeding 100% of the predicted maximum heart rate, and an RPE of 19 or higher, despite verbal encouragement. The duration that each participant was able to continue before reaching their limit of exhaustion was documented as the time to exhaustion for every participant (Machado et al. 2013).

### 2.8 RPE

The Borg scale, which measures perceived exertion on a range from 6, indicating no exertion at all, to 20, representing maximal exertion, was utilized to assess the RPE of the participants (Astorino et al. 2012). Throughout the duration of the submaximal running test, participants were instructed to report their RPE at intervals of every 3 min, as well as at the point of exhaustion. The responses provided by the participants were then used to calculate the mean RPE, which formed the basis for subsequent statistical analysis. In the initial familiarization session, participants learned about RPE and how to report their responses at specified intervals.

### 2.9 Affective Valence and Perceived Activation

Affective valence (pleasure‐displeasure) was assessed using the Feelings Scale (FS), consisting of 11 items on a range from −5 (very negative) to +5 (very positive), with zero indicating a neutral effect (Etemadi et al. 2023). Arousal levels were measured with the Felt Arousal Scale (FAS), comprising six items scored from 1 (low arousal) to 6 (high arousal) (Astorino et al. 2012). During the initial familiarization session, participants were instructed on affective valence, arousal, and how to report their perceptual responses at set intervals during the submaximal running test. The responses from these scales were used to calculate the mean affective valence and arousal for statistical analyses.

### 2.10 CRT

The CRT is a key indicator of cognitive performance and is essential for athletic performance (Erickson 2021; Nakamoto and Mori 2008). We measured CRT using an apparatus (Model 63,035 A, Lafayette Instrument Company, Indiana, USA) previously utilized in our laboratory for reaction time assessments under normobaric hypoxia (Etemadi et al. 2023). A four‐choice stimulus–response paradigm was employed, where participants sat comfortably in front of a response panel with four lights and corresponding buttons. Three visual stimuli (lights turning on) were presented manually, and participants were instructed to respond as quickly as possible by pressing the corresponding button. The reaction time for each stimulus was recorded, and the mean value of the three trials was calculated as the final CRT score. The CRT test was conducted at the beginning of each experimental session before hypoxic exposure, 60 min after hypoxic exposure, and immediately after exhaustion in the endurance task under hypoxia.

### 2.11 SCWT

The SCWT assesses inhibitory control, defined as the capacity to manage attention, behavior, thoughts, and emotions to resist automatic (impulsive) actions and achieve specific goals (Diamond 2013). The Stroop test (ST) is a reliable and valid method for assessing inhibitory control, which is vital for regulating intense physical activities by suppressing unpleasant sensations during exercise (Angius et al. 2019).

This study utilized software to conduct the SCWT, which is based on variables from the ST (Khodadadi et al. 2014). In the ST, participants view 48 congruent and 48 incongruent colored words in red, blue, yellow, and green, where the word's color matches its meaning (e.g., “green” in green). Incongruent words have mismatched colors (e.g., “green” in red, blue, or yellow). The 96 words are presented in random order, with each stimulus displayed for 2 s and an 800‐ms interval between stimuli. Participants must identify the words' colors, ignoring their meanings (Khodadadi et al. 2014). Interference time was calculated by subtracting the mean reaction time for congruent trials from the mean reaction time for incongruent trials and was expressed in milliseconds. Interference score was calculated by subtracting the number of correct responses to incongruent trials from the number of correct responses to congruent trials. Higher positive values indicated greater interference in response time and response accuracy, respectively. Research supports the Stroop test's reliability and validity in measuring inhibition in both adults and children, with test–retest reliability reported between 0.80 and 0.91 (Baron 2018; MacLeod 1991). The SCWT was administered at the start of each session under normoxic conditions, 60 min after hypoxic exposure, and after reaching exhaustion in hypoxia.

### 2.12 HR and SpO_2_

The HR was continuously monitored using a chest strap (Polar, Finland) while SpO_2_ levels were also constantly monitored by a fingertip pulse oximeter (Nonin Onyx II 9550, Nonin Medical, Plymouth, MN, USA). Participants' HR and SpO_2_ were recorded at 3‐min intervals during the submaximal running test to exhaustion, and mean values were calculated for statistical analysis.

### 2.13 Normobaric Hypoxia Exposure

The hypoxic air apparatus (GO2Altitude ERA II, Bio MedTech, Melbourne, Australia) generated the target normobaric hypoxic condition (O_2_ = 13%, equivalent to an altitude of ~3800 m). It continuously pumped hypoxic air into two 120‐L Douglas bags. During each experimental session, participants donned a comfortable inflatable air cushion mask with a non‐rebreathing valve, secured by a rubber port full‐face harness, which was connected to the Douglas bags.

### 2.14 Statistical Analysis

The normal distribution of each data set was evaluated by the Shapiro–Wilk normality test. Data are presented as mean ± SD. Because the Stroop interference score did not meet the assumption of normality, it was analyzed using the Friedman test, followed by Wilcoxon signed‐rank tests for pairwise comparisons. CRT and interference time of the ST were analyzed using a two‐way repeated measures ANOVA (2 × 3 factorial design) to examine the effects of supplementation condition (GB vs. placebo), time (rest under normoxia, hypoxia, and post‐exhaustion in hypoxia), and their interaction. A paired *t*‐test compared endurance performance and physiological and psychophysiological variables (TTE, HR, SpO_2_, RPE, FS, and FAS) between GB and placebo supplementations. Effect sizes for the ANOVAs were measured using partial eta squared (*ɳ* ^2^_p_), interpreted as small (0.01–0.059), medium (0.06–0.139), or large (≥ 0.14). Cohen's *d* was used for the paired *t*‐test effect size, classified as small (0.20–0.49), medium (0.50–0.79), or large (≥ 0.80). Participants' paired guesses during the actual GB and placebo phases were compared using an exact marginal homogeneity test. All statistical analyses were performed using SPSS version 26 (SPSS Inc., Chicago, IL, USA), with a significance level of *p* < 0.05.

## 3 Results

### 3.1 Participant Characteristics

The descriptive characteristics of the participants are shown in Table 1.

**TABLE 1:** Descriptive characteristics of the participants.

Variable	Mean ± SD
Age (years)	37.0 ± 5.2
Height (cm)	178.9 ± 6.4
Body mass (kg)	73.9 ± 8.3
BMI (kg/m^2^)	23.0 ± 1.7

*Note:* Data are presented as mean ± standard deviation.

### 3.2 Assessment of Participant Blinding

Participants' guesses did not differ significantly between the actual GB and placebo phases (exact marginal homogeneity test, *p* = 0.766). Three participants correctly identified both conditions, three identified both conditions incorrectly by reversing the two conditions, and six were uncertain in at least one phase. These findings provided no clear evidence that participants could distinguish the GB and placebo conditions.

### 3.3 Endurance Performance and Physiological Responses

**FIGURE 2:**
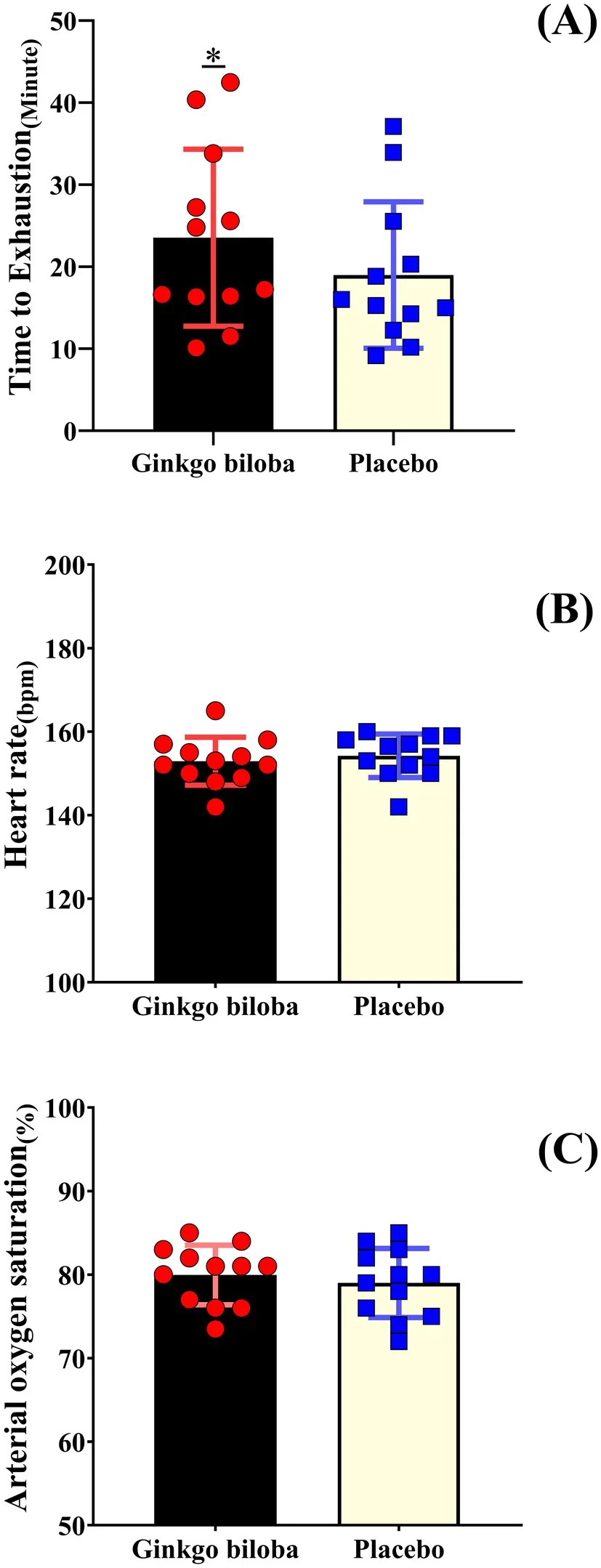
Mean values of time to exhaustion under *Ginkgo biloba* and Placebo conditions under hypoxia (A). Mean values of heart rate (B) and arterial oxygen saturation (C) during the endurance task under *G. biloba* and Placebo conditions under hypoxia (*significant difference between conditions).

*G. biloba* (GB) supplementation significantly increased time to exhaustion (TTE) under hypoxic conditions compared with placebo (23.53 ± 10.8 vs. 18.98 ± 8.9 min, *t*_11_ = 2.738, *p* = 0.019, *d* = 0.79) (Figure 2A). In contrast, no significant between‐condition differences were observed for heart rate (HR) (152.92 ± 5.7 vs. 154.21 ± 5.2 bpm, *t*_11_ = −0.450; *p* = 0.661, *d* = 0.13) (Figure 2B) or blood oxygen saturation (SpO_2_) during the exhaustion test (79.96% ± 3.6% vs. 79.00% ± 4.1%, *t*_11_ = 1.013, *p* = 0.333, *d* = 0.29) (Figure 2C).

### 3.4 Psychophysiological Responses

**FIGURE 3:**
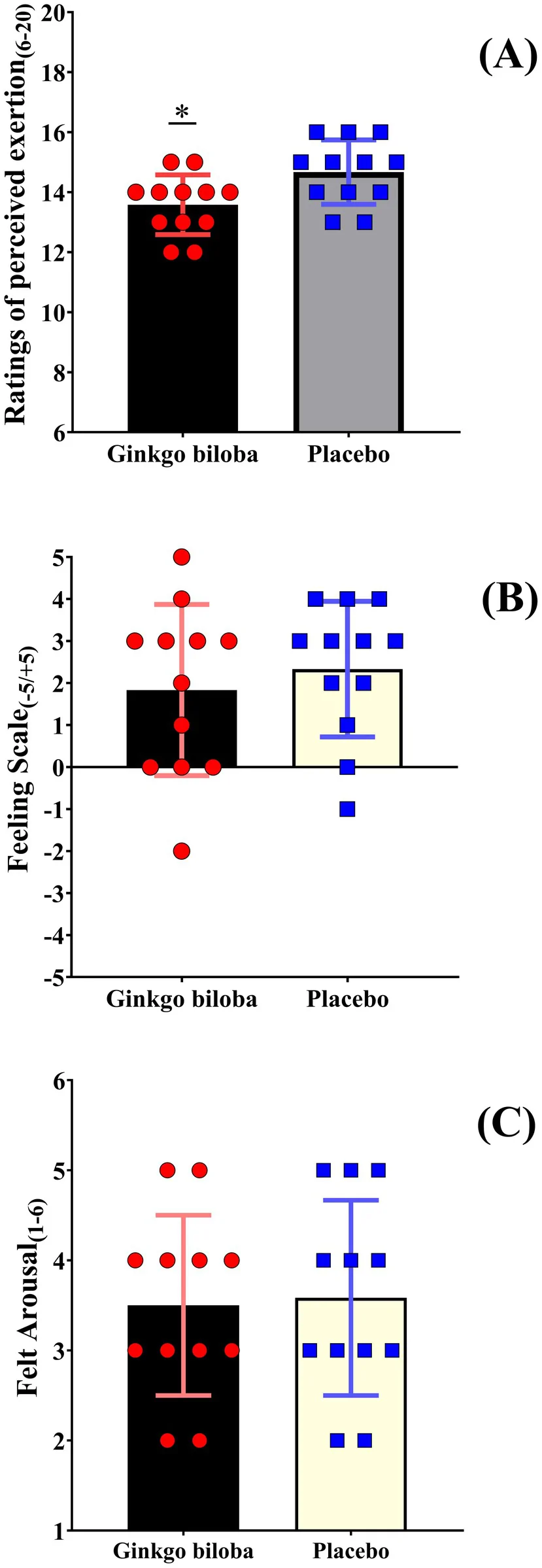
Mean values of ratings of perceived exertion (A), feeling of pleasure (B), and felt Arousal (C) during the endurance task under *Ginkgo biloba* and Placebo conditions under hypoxia (*significant difference between conditions).

GB supplementation significantly reduced rate of perceived exertion (RPE) during the exhaustion test compared with placebo (13.58 ± 1.0 vs. 14.67 ± 1.1, *t*_11_ = −2.721, *p* = 0.020, *d* = 0.78) (Figure 3A). However, no significant between‐condition differences were observed for FS scores (1.83 ± 2.0 vs. 2.33 ± 1.6, *t*_11_ = −1.318, *p* = 0.214, *d* = 0.38) (Figure 3B) or Felt Arousal Scale (FAS) scores (3.46 ± 1.0 vs. 3.58 ± 1.1, *t*_11_ = −0.583, *p* = 0.571, *d* = 0.17) (Figure 3C).

### 3.5 Cognitive Performance

No statistically significant between‐condition differences were detected in choice reaction time (CRT). Repeated‐measures ANOVA showed no significant interaction effect between supplementation and time (*F*_2,22_ = 1.751, *p* = 0.197, *ɳ* ^2^_p_ = 0.137), no significant main effect of supplementation (*F*_1,11_ = 1.163, *p* = 0.304, *ɳ* ^2^_p_ = 0.096), and no significant main effect of time (*F*_2,22_ = 1.757, *p* = 0.196, *ɳ* ^2^_p_ = 0.138) (Table 2).

**TABLE 2:** The results of cognitive performance at rest under normoxia, hypoxia, and post exhaustion in hypoxia.

Variable	Condition	Mean ± SD	p	Mean ± SD	*p*	Mean ± SD	*p*
		Rest	Rest	Hypoxia	Hypoxia	TTE	TTE
CRT score	GB	0.37 ± 0.1	0.722	0.36 ± 0.1	0.090	0.34 ± 0.1	0.978
	Placebo	0.36 ± 0.1		0.33 ± 0.1		0.34 ± 0.1	
IT score	GB	47.08 ± 43.7	0.889	45.08 ± 44.60	0.608	46.50 ± 45.20	0.943
	Placebo	45.08 ± 48.7		54.33 ± 55.00		47.25 ± 29.90	
IS score	GB	0.42 ± 1.7	0.304	0.42 ± 2.1	0.796	0.50 ± 1.6	0.812
	Placebo	0.92 ± 1.4		0.42 ± 1.4		0.25 ± 2.0	

*Note:* Data are presented as mean ± standard deviation.

Similarly, no statistically significant between‐condition differences were detected in Stroop Color‐Word Test (SCWT) outcomes. For Stroop interference time (IT), repeated‐measures ANOVA showed no significant interaction effect (*F*_2,22_ = 0.191, *p* = 0.827, *ɳ* ^2^_p_ = 0.017), no significant main effect of supplementation (*F*_1,11_ = 0.086, *p* = 0.775, *ɳ* ^2^_p_ = 0.008), and no significant main effect of time (*F*_2,22_ = 0.107, *p* = 0.899, *ɳ* ^2^_p_ = 0.010). For Stroop interference scores (IS), Friedman tests showed no significant differences across rest under normoxia, hypoxia, and post‐exhaustion in hypoxia in either the GB condition (*X* ^2^ = 1.167, *p* = 0.558) or the placebo condition (*X* ^2^ = 2.811, *p* = 0.245). Wilcoxon signed‐rank tests also showed no significant between‐condition differences in IS at rest under normoxia (*Z* = −1.029, *p* = 0.304), during hypoxia (*Z* = −0.259, *p* = 0.796), or after exhaustion under hypoxia (*Z* = −0.238, *p* = 0.812) (Table 2).

## 4 Discussion

The present study investigated the effects of GB supplementation, used as an adjunct to Az for AMS prevention, on exercise and cognitive performance in male mountaineers under hypoxic conditions. The main finding was a longer time to exhaustion during the GB phase than in the placebo phase, whereas no significant between‐condition differences were observed in the cognitive outcomes.

The longer time to exhaustion during the GB phase was accompanied by lower RPE during the exhaustion test. This pattern is broadly in line with a previous human study that reported improved aerobic performance after GB supplementation in physically active men (Sadowska‐Krępa et al. 2017). However, not all previous findings have been consistent. For example, Wang et al. (2007) did not observe an added benefit of GB on walking capacity or submaximal oxygen consumption in patients with peripheral arterial disease, which may reflect important differences in participant characteristics, clinical status, exercise protocol, and study context compared with the present sample (Wang et al. 2007). In the present study, HR and SpO_2_ were similar between conditions despite the longer time to exhaustion and lower mean RPE during the GB phase. The current measurements therefore provide no evidence that the longer exercise tolerance was accompanied by a different mean heart‐rate response or improved arterial oxygen saturation. Instead, this pattern is compatible with a perceptual or effort‐related contribution, such as a lower perception of effort or a greater tolerance of effort at the prescribed workload. Participants' condition guesses provided no clear evidence that they could distinguish GB from placebo, reducing, although not eliminating, the likelihood that the lower RPE reflected differential treatment expectations. Recent experimental studies have shown that differences in endurance tolerance can occur alongside differences in perceived effort despite similar commonly measured cardiorespiratory or metabolic responses (Díaz‐Lara et al. 2024; Lopes et al. 2020). Antioxidant, anti‐inflammatory, and vasoactive actions of GB have also been described in previous reviews (Akaberi et al. 2023; Zonouz et al. 2024), and some exercise‐related evidence suggests that GB supplementation may attenuate post‐exercise increases in creatine kinase and C‐reactive protein (Atashak et al. 2021). These observations identify potential pathways for future investigation, but they do not establish the mechanism underlying the present finding because these pathways were not assessed in this study. Oxygen uptake, ventilation, blood lactate, muscle or cerebral oxygenation, and neuromuscular fatigue were also not measured. An unmeasured physiological contribution therefore cannot be excluded, and the mechanism underlying the longer time to exhaustion remains uncertain. Because Az was administered in both conditions, the findings reflect the effect of adding GB during Az use rather than the effect of GB alone. The present design therefore does not show whether the same response would have occurred without Az. This point is relevant because recent evidence suggests that carbonic anhydrase inhibition itself can influence exercise performance during acute hypoxic exposure (Chang et al. 2024). Although the increase in time to exhaustion reached statistical significance, its practical relevance for real‐world high‐altitude performance remains uncertain. Given the modest sample and the number of outcomes examined, this finding should be regarded as exploratory and requires confirmation in a larger study.

No statistically significant between‐condition differences were detected in CRT or Stroop performance in the present study. This result is not necessarily inconsistent with the broader literature. While previous reviews have pointed to possible neuroprotective effects of GB in certain cognitive and neurological conditions (Singh et al. 2019; Zonouz et al. 2024), evidence from human studies remains heterogeneous and appears to depend, at least in part, on the population studied and the cognitive domain assessed (Lorca et al. 2023). The present findings should also be interpreted in light of the hypoxic context itself, as acute hypoxic exposure has been shown to impair several aspects of cognitive performance, including attention, memory, reaction time, and response accuracy (Chen et al. 2023; Ramírez‐delaCruz et al. 2024). A further consideration is the concurrent use of acetazolamide. Earlier studies associated acetazolamide with poorer cognitive performance shortly after ascent (Wang et al. 2013), and Phillips et al. (2017) reported similar negative cognitive effects in individuals traveling to high altitude while taking the drug (Phillips et al. 2017). However, more recent work suggests that the cognitive effects of acetazolamide at altitude may be more complex and not uniformly detrimental under all circumstances (Reiser et al. 2023). Accordingly, it is possible that any cognitive effect of GB in the present study was too subtle to detect, not adequately captured by the selected tasks, or diminished by the short supplementation period and concomitant Az use. These findings should be interpreted cautiously. Although the crossover design reduced between‐participant variability, the modest sample size limited the precision of the cognitive estimates, particularly for the more variable Stroop measures. The relatively large SDs for the interference score mainly reflect the way this measure was calculated as a signed difference between correct responses in congruent and incongruent trials. Because individual scores fell on both sides of zero, positive and negative values produced group means close to zero while the SD remained similar to or larger than the mean. The present findings therefore do not support a clear cognitive benefit of GB under these conditions, but smaller or less consistent effects cannot be excluded.

## 5 Limitations

Several limitations should be considered when interpreting the present findings. First, the study was conducted under controlled normobaric hypoxic conditions, which allowed for greater experimental control but did not fully reflect the environmental and physiological demands of real high‐altitude exposure, such as cold, cumulative fatigue, and changes in barometric pressure. Second, the supplementation period was relatively short, and this may have reduced the likelihood of detecting subtle cognitive effects of GB. Third, despite the crossover design, the modest sample size limited the study's ability to detect smaller cognitive effects, particularly given the variability in the Stroop measures. In addition, the sample was restricted to male mountaineers, which further limits extrapolation to other populations. All participants were regularly aerobically active, which may have influenced their baseline exercise tolerance and their physiological, perceptual, or cognitive responses to acute hypoxia. Although the crossover design and standardized testing procedures reduced the influence of training status on the between‐condition comparison, the findings may not generalize to sedentary or less‐trained individuals. Finally, while a washout period was included, residual treatment effects and possible learning effects related to repeated cognitive testing cannot be entirely excluded.

## 6 Conclusion

In conclusion, GB supplementation at 200 mg/day for 5 days was associated with a longer time to exhaustion at a simulated altitude of 3800 m in male mountaineers using Az for AMS prevention. No significant between‐condition differences were observed in cognitive outcomes. Because Az was administered in both conditions, these findings apply to GB used alongside Az and do not establish the effect of GB when used alone. Given the modest sample, the endurance finding should be regarded as exploratory and requires confirmation in larger studies, particularly under real high‐altitude conditions.

## Author Contributions

**Sara Mansouri:** writing – original draft, conceptualization, methodology, data curation, investigation, validation. **Vahid Tadibi:** writing – review and editing, visualization, validation, methodology, writing – original draft, funding acquisition, investigation, conceptualization, project administration, formal analysis, software, data curation, supervision, resources.

## Funding

The authors have nothing to report.

## Ethics Statement

The study was conducted in accordance with the Declaration of Helsinki and approved by the Research Ethics Committees of Kermanshah Razi University (IR.RAZI.REC.1402.014).

## Conflicts of Interest

The authors declare no conflicts of interest.

## Data Availability

The data generated and/or analyzed during the current study are available from the corresponding author upon reasonable request.
